# Cyclin D1 expression in penile cancer

**DOI:** 10.18632/oncotarget.28584

**Published:** 2024-05-14

**Authors:** Wesliany Everton Duarte, Jaqueline Diniz Pinho, Syomara Pereira da Costa Melo, Denner Rodrigo Diniz Duarte, Juliana Martins da Guia Ribeiro do Carmo, André Salim Khayat, José Ribamar Rodrigues Calixto, Marcos Adriano Garcia Campos, Rita da Graça Carvalhal Frazão Correa, Antonio Machado Alencar Júnior, Antônio Augusto Lima Teixeira-Júnior, Gyl Eanes Barros Silva

**Affiliations:** ^1^Postgraduate Program in Adult Health, Federal University of Maranhão, Maranhão, Brazil; ^2^Clinical Research Center, University Hospital of the Federal University of Maranhão, São Luís, Brazil; ^3^State University of Maranhão, Zé Doca, Brazil; ^4^University Hospital, Federal University of Maranhão, São Luís, Brazil; ^5^Oncology Research Center, Federal University of Pará, Belém, Brazil; ^6^Clinical Hospital of Medical School, São Paulo, State University, Unesp, Botucatu, São Paulo, Brazil; ^7^Postgraduate Program in Nursing, Federal University of Maranhão, Maranhão, Brazil; ^8^Postgraduate Program in Health Science, Federal University of Maranhão, Maranhão, Brazil; ^9^Department of Genetics and Postgraduate Program in Genetics, Ribeirão Preto Medical School, University of São Paulo, Ribeirão Preto, Brazil; ^10^Department of Pathology, Ribeirão Preto Medical School, University of São Paulo, Ribeirão Preto, Brazil

**Keywords:** immunohistochemistry, biomarkers, cyclin D1, penile neoplasms

## Abstract

The main goal of the present study was to analyze the expression profile of cyclin D1 in patients with PC, and to determine possible correlations with clinical and histopathological features. A survey was conducted with 100 patients diagnosed with PC, who were treated at two reference hospitals in São Luís, Maranhão, Brazil, between 2013 and 2017. A review of clinical, epidemiological, and histopathological data was performed, Human Papillomavírus (HPV) DNA was detected using polymerase chain reaction (PCR) and cyclin D1 expression analysis was performed using immunohistochemical techniques. The data revealed that the absence of cyclin D1 expression was significantly associated with HPV-positive histological subtypes (*p* = 0.001), while its expression was associated with high-grade tumors (*p* = 0.014), histological subtype (*p* = 0.001), presence of sarcomatoid transformation (*p* = 0.04), and perineural invasion (*p* = 0.023). Patients with cyclin D1 expression exhibited lower disease-free survival compared to the cyclin D1-negative group, although the difference was not statistically significant. The results suggest that cyclin D1 may be a potential biomarker for PC, especially for poorer prognosis.

## INTRODUCTION

Penile cancer (PC) is a malignant neoplasm that frequently affects males in their sixth decade of life with low socioeconomic status and educational level [1]. This is an uncommon disease in developed countries, but highly incident in developing nations. Brazil holds the global record for PC incidence [2]. The etiology of PC is heterogeneous and remains under investigation; however, some risk factors, such as phimosis, poor hygiene, alcoholism and smoking, and human papillomavirus (HPV) infection, have been proposed [1–4].

Among these risk factors, HPV infection appears to play a role in approximately one-half of all cases. The mechanism of HPV action in PC appears to be similar to that in cervical cancer, in which the oncoproteins E6 and E7 act by inactivating the suppressor proteins p53 and retinoblastoma (pRb), respectively [5]. These oncoproteins directly impact control of the cell cycle, which leads to dysregulation in cell proliferation. When the E7 oncoprotein binds pRb, it triggers the release of the E2F transcription factor, which then induces cell cycle progression [6]. In several tumors, this mechanism triggers a cellular response frequently related to p16 overexpression and decrease in cyclin D1 expression [7, 8].

Cyclin D1 is a protein encoded by the *CCND1* gene and acts in the G_1_/S phase of the cell cycle, positively regulating cell proliferation and differentiation [8]. This protein has been widely studied due to its important role in cell cycle regulation and biochemistry, both in normal and tumor cells [9]. Overexpression of cyclin D1 can lead to shortening of the G_1_ phase, reducing dependence on mitogenic factors and causing increased cell proliferation. Thus, it has been proposed that this mechanism may be involved in cell transformation and tumorigenesis [10].

Several studies have demonstrated changes in cyclin D1 expression, both in the early stage and along tumor progression, in different types of cancers, including breast [10], head and neck [11], prostate [12] and renal cell carcinoma [13] and PC [14]. These findings suggest that cyclin D1 may be a potential biomarker for cancer.

Regarding PC, however, few studies have assessed the role of cyclin D1, reinforcing the necessity for initiatives that aim to investigate its actual role in the pathophysiology of this disease. As such, the present study aimed to characterize the expression of cyclin D1 in patients with PC, and to determine possible correlations with the clinical and histopathological features of the disease.

## RESULTS

### Clinical profile of patients with PC

The mean age of the study cohort was 60.1 years (range, 26 to 93 years), with the majority being ≤60 years of age (46.0%). These patients had a low educational level (99.0%), the majority were farmers (66.5%) with poor hygiene habits (72.5%) and phimosis (67.5%). Risky sexual behavior was also described, in which 70% of patients reported to have >6 sexual partners through life and 58% reported engaging in sex with animals (i.e., zoophilia). Clinical and epidemiological information is summarized in Table 1.

**Table 1:** Clinical-epidemiological features of 100 patients with penile cancer

Variables	*N* (%)
**Age group**	
18---| 40 years	18 (18)
41---| 60 years	28 (28)
>60 years	54 (54)
**Educational level (*N* = 71)**	
Uneducated or incomplete elementary school	59 (83)
Elementary school or incomplete high school	11 (16)
High school or complete/incomplete higher education	1 (1)
**Main occupation (*N* = 93)**	
Farmer	62 (66.5)
Retired	12 (13)
Others	19 (20.5)
**Smoker (*N* = 81)**	
No	38 (47)
Yes	43 (53)
**Alcoholic (*N* = 54)**	
No	25 (46)
Yes	29 (54)
**Phimosis (*N* = 77)**	
No	25 (32.5)
Yes	52 (67.5)
**Zoophilia (*N* = 50)**	
No	21 (42)
Yes	29 (58)
**Genital hygiene (*N* = 58)**	
Good hygiene	16 (27.5)
Poor/moderated hygiene	42 (72.5)
**Number of sexual partners (*N* = 47)**	
<6 partners	14 (30)
6–10 partners	8 (17)
>10 partners	25 (53)
**Beginning of symptoms (*N* = 64)**	
0 to 12 months	40 (62.5)
>12 months	24 (37.5)

### Histopathological features

The primary surgery performed was partial amputation of the penis (73%), with lesions predominantly located on the glans (98%). All tumors were classified as squamous cell carcinoma with prevalence of the usual subtype (35.3%). When analyzing the subtypes according to HPV-associated or non-HPV-associated etiology, there was a prevalence of HPV-associated tumors (65%), especially warty, basaloid, warty-basaloid, and mixed. Most tumors were high-grade (G3 (57%)); pT3/pT4 (61%); and stage II (76%)). Approximately 44 patients underwent inguinal lymphadenectomy, of whom 28 (63.5%) had lymph node metastasis confirmed in the histopathological diagnosis. The presence of changes suggestive of HPV infection (i.e., koilocytosis) was observed in 87% of cases. A summary of the histopathological data is presented in Table 2.

**Table 2:** Clinical histopathological features of 100 patients with penile cancer

Variables	*N* (%)
**Topography**	
Restricted to foreskin/balanopreputial groove	2 (2)
Glans and foreskin/Glans and groove	67 (67)
Glans and body/Body	31 (31)
**Size of lesion (*N* = 99)**	
0.6–2.0 cm	7 (7.1)
2.1–5.0 cm	55 (55.5)
5.1–10 cm	37 (37.4)
**Histological subtype (*N* = 99)**	
Basaloid	6 (6.1)
Warty	29 (29.3)
Usual	35 (35.3)
Wart-basaloid	8 (8.1)
Mixed	19 (19.2)
Others^*^ (medullary and pseudo-hyperplastic)	2 (2)
**Differentiation grade**	
G1	13 (13)
G2	30 (30)
G3	57 (57)
**Perineural invasion**	
No	62 (62)
Yes	38 (38)
**Corpus cavernosum (*N* = 99)**	
Free	41 (41.4)
Compromised	58 (58.6)
**Corpus spongiosum**	
Free	16 (16)
Compromised	84 (84)
**Urethra**	
Free	25 (25)
Compromised by contiguity	15 (15)
Compromised	59 (59)
**Tumoral focus**	
Unifocal	85 (85)
Multifocal	15 (15)
**Presence of carcinoma *in situ***	
No	17 (17)
Yes	83 (83)
**Sarcomatoid transformation**	
No	79 (79)
Yes	21 (21)
**Lymphocytic infiltrate (*N* = 99)**	
Absent	6 (6.1)
Present	93 (93.9)
**Primary tumor**	
pT1	14 (14)
pT2	25 (25)
pT3/pT4	61 (61)
**Staging**	
I	13 (13)
II	76 (76)
III–IV	11 (11)
**Surgery type**	
Partial penectomy	73 (73)
Total penectomy	27 (27)
**Lymph node metastasis (*N* = 44)**	
Absent	16 (36.4)
Present	28 (63.6)
**Extra node extension (*N* = 44)**	
Absent	20 (45.5)
Present	24 (54.5)
**Koilocytosis**	
Absent	13 (13)
Present	87 (87)
**Invasion pattern (*N* = 97)**	
Expansive	76 (78.5)
Infiltrative	21 (21.5)
**HPV DNA (*N* = 97)**	
HPV-Positive	63 (65)
HPV-Negative	34 (35)

Others^*^ = whitish, whitish with poorly defined edges, fistulous, necrotizing, brownish and purulent.

### Cyclin D1 expression

**Figure 1:**
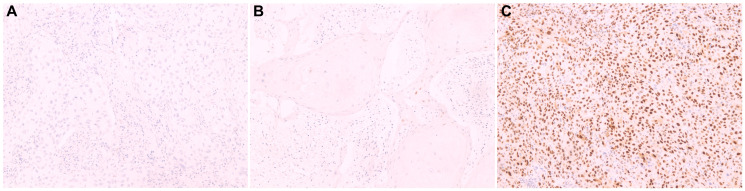
Cyclin D1 protein expression by immunohistochemistry in histological sections. (**A**) Negative penile squamous cell carcinoma, showing absence of staining in the basal layer cells; (**B**) Negative penile squamous cell carcinoma showing weak staining in basal layer cells; (**C**) Positive penile squamous cell carcinoma, with staining in the suprabasal layers. Magnification 100x (A-O).

Analysis of the cyclin D1 expression profile revealed that most cases exhibited weak or no immunostaining (56%), which was considered to be negative for cyclin D1 expression (Figure 1). Statistical analysis revealed that the absence of cyclin D1 expression was associated with tumors with HPV-related histological etiology (*p* = 0.001), while its expression was associated with high-grade tumors (G3, *p* = 0.0014), sarcomatoid transformation (*p* = 0.04), perineural invasion (*p* = 0.023), and histological subtype (*p* = 0.001). Data regarding associations between cyclin D1 expression profile and clinical and histopathological features are presented in Table 3.

**Table 3:** Association of cyclin D1 regarding clinical-histopathological features

Variables	Cyclin D1		*p*-value^*^
	Negative (−)	Positive (+)	
**Phimosis (*N* = 77)**			
Absent	11	14	0.6
Present	20	32	
**Surgery type**			
Glansectomy/partial penectomy	29	44	0.17
Total penectomy	15	12	
**Size of lesion (*N* = 99)**			
0.6–5.0 cm	25	37	0.53
5.1–10 cm	18	19	
**HPV-associated histological subtype (*N* = 81)**			
Non-associated	4	33	0.001
Associated	23	21	
**Differentiation grade**			
G1/G2	18	25	0.014
G3	11	46	
**Perineural invasion**			
No	23	39	0.023
Yes	6	32	
**Tumoral focus**			
Unifocal	35	50	0.25
Multifocal	9	6	
**Presence of carcinoma *in situ***			
No	6	11	0.59
Yes	38	45	
**Sarcomatoid transformation**			
No	39	40	0.04
Yes	5	16	
**Lymph node infiltrate (*N* = 99)**			
Absent	4	2	0.39
Present	39	54	
**Primary tumor**			
pT1 and pT2	18	21	0.83
pT3 and pT4	26	35	
**Staging**			
I–II	38	51	0.52
III–IV	6	5	
**Lymph node metastasis (*N* = 44)**			
Absent	6	10	1
Present	11	17	
**Extra node extension (*N* = 43)**			
Absent	7	10	0.76
Present	12	14	
**Koilocytosis**			
Absent	1	12	0.10
Present	28	59	
**Invasion pattern (*N* = 97)**			
Expansive	23	53	0.14
Infiltrative	3	18	
**Histological subtype (*N* = 98)**			
Basaloid	5	1	0.001
Warty	14	15	
Usual	3	32	
Warty-basaloid	4	5	
Mixed	2	17	
Others	1	1	

### Disease-free survival analysis

**Figure 2:**
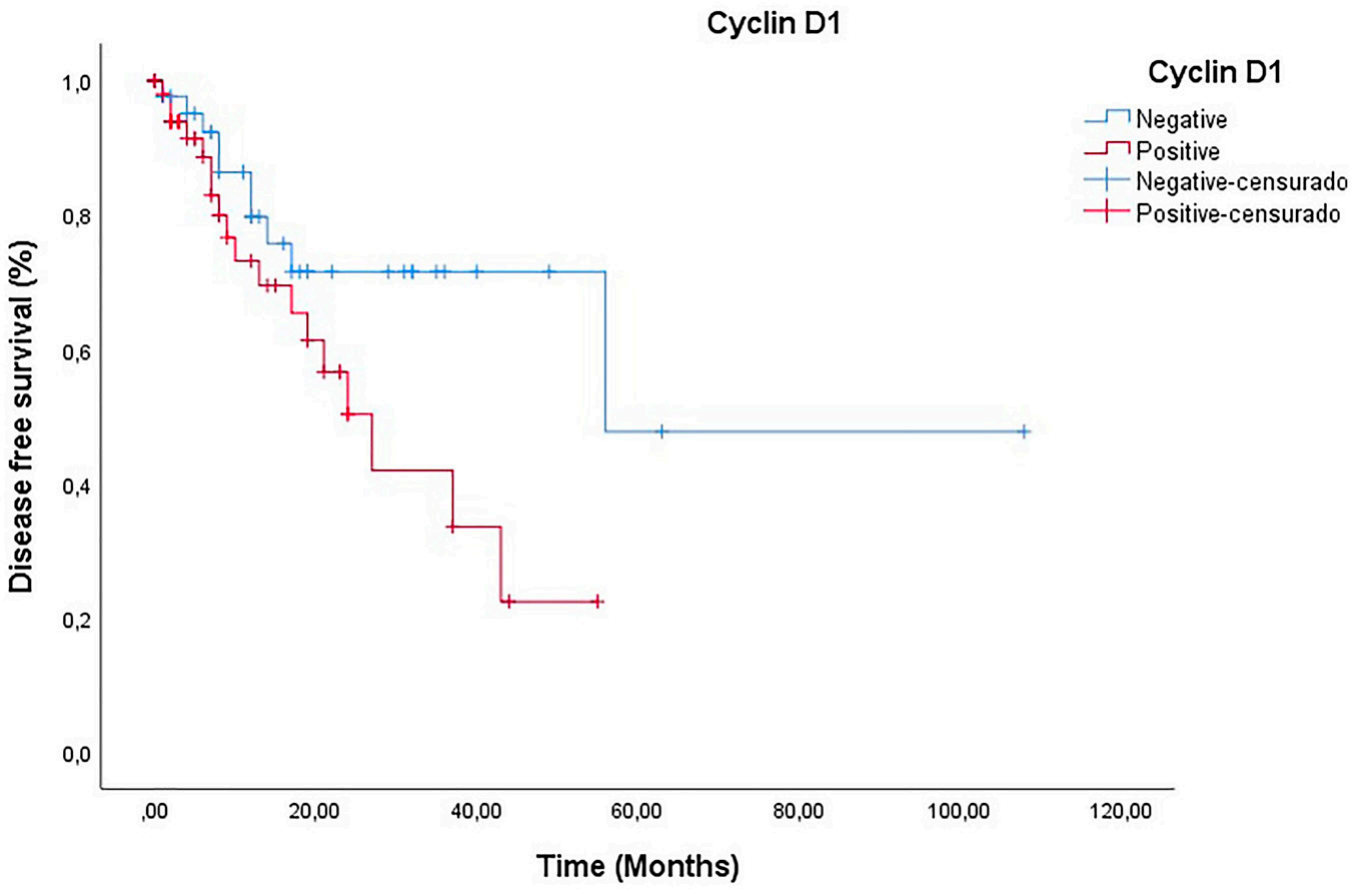
Analysis of disease-free survival of patients according to the presence and absence of cyclin D1 (Log-Rank *p* = 0.669).

The data revealed that patients with PC and positive expression of cyclin D1 had a shorter disease-free survival (28.5 months) compared to those who did not express cyclin D1 (67.6 months), although the difference was not statistically significant (log-rank *p* = 0.669) (Figure 2).

## DISCUSSION

The state of Maranhão, Brazil, registers the highest global incidence of PC, and has the worst Human Development Index (0.639) [1, 2, 4, 15]. In our sample, there was a predominance of patients younger than their seventh decade of life, with low educational level, poor hygienic conditions, and phimosis, as well as smokers and those who engaged in zoophilia. These data reinforce the relationship between PC and a low socioeconomic profile. Due to the advanced stage of disease in virtually all cases, the treatment was penile amputation, which has physical, social, and psychological implications for patients. As such, PC is considered to be a public health problem in many underdeveloped countries.

Therefore, one of the current targets of scientific research has been biomarkers that provide the possibility of aiding prognosis and therapy, while also being capable of identifying the main molecular agents involved in the tumorigenic process. Studies have shown that high expression of cyclin D1 in head and neck, colorectal, esophageal and breast tumors have been considered to be a characteristic of poorer prognosis, thus becoming a potential biomarker [11, 16, 17]. In our study, the expression of this protein was significantly associated with the following clinical features: histological subtype associated with HPV; degree of differentiation; sarcomatoid transformation; perineural invasion; and pattern of infiltrative invasion.

The absence of cyclin D1 expression was associated with the HPV-positive histological subtype. Corroborating our results, in head and neck, oropharyngeal and cervical cancers associated with HPV, cyclin D1 was down-regulated [11, 16, 18]. It has been well described that in HPV-associated tumors, the HPV oncoprotein E7 is responsible for the increase in p16 expression levels. It is also known that p16 is a tumor suppressor protein that acts by inhibiting the cyclin D1/CDK4 complex, favoring the survival of tumor cells [19]. Thus, overexpression of p16 leads to the absence of cyclin D1 expression and, consequently, of the p16INK4a/cyclinD/Rb pathway [20].

Cyclin D1 expression was more frequent in tumors with an infiltrative invasion pattern. This type of tumor is characterized by its rapid and disordered growth, with dissemination to nearby structures, presenting a greater risk for nodal involvement [21]. In basal cell and basosquamous carcinomas, cyclin D1 positivity was identified as a marker of aggressive behavior [22]. Furthermore, in prostate cancer, the expression of cyclin D1 appears to increase the capacity for tumor invasion, reinforcing the involvement of this protein in the more aggressive behavior of some tumors [12, 23].

Cyclin D1 expression was also associated with histological grade G3 and sarcomatoid transformation. Tumors with sarcomatoid areas must always be considered as G3, regardless of the ratio. Poorly differentiated tumors are characterized by greater aggressiveness, indicating faster growth compared to those that are well differentiated [24]. These data corroborate the findings in esophageal and breast cancer, in which the expression of cyclin D1 was associated with poorly differentiated tumors [10, 25]. Tumors with sarcomatoid alterations are more prone to lymph node metastasis, thus portending a poorer prognosis.

The presence of perineural invasion is associated with cyclin D1 expression. Studies have reported that the presence of this protein in cases of perineural invasion is considered to be a characteristic of poorer clinical prognosis because this condition is characterized by increased local recurrence and regional metastasis [26, 27]. It is believed that perineural invasion is factor-mediated by growth factors and that their receptors interact with cyclin D1.

In breast and oropharyngeal tumors, the positive expression of cyclin D1 was associated with lower disease-free survival [10, 28]. Thus, our results corroborate those described in the literature for cancer in general, in which patients who exhibited expression of this protein exhibited worse disease-free survival [27, 29]. The increased expression of this protein may be related to dysregulation in its degradation because this process is important for DNA replication.

To our knowledge, there are only two studies that have investigated the role of cyclin D1 in the genesis and behavior of PC. The first, by Papadopoulos et al. [30], reported the overexpression of cyclin D1 in 61.9% of assessed cases of PC, being more frequent in poorly differentiated tumors with a high proliferative index, although without statistical significance. The second, by Gunia et al. [14], evaluated the expression of cyclin D1 in 110 penile tumors. This study also found no association between the expression profile and the parameters studied. The results reported in these two studies are divergent from ours, as cyclin D1 appeared to demonstrate prognostic relevance. However, we emphasize the need for further studies to validate this protein as a biomarker in PC.

A limitation of our study was the lack of information in medical records, as well as the absence of follow-up for some patients. Furthermore, only 100 cases were selected due to the poor condition of the paraffin blocks, making it exceedingly difficult to perform immunohistochemical analysis with a larger sample. Nevertheless, due to the rarity of the disease, the number of cases in our study still makes a significant contribution to the understanding of this pathology.

## MATERIALS AND METHODS

### Study design

The research was performed from 2013 to 2017, and included 100 patients with PC treated at two reference hospitals in São Luís, Maranhão, Brazil (University Hospital of the Federal University of Maranhão - HUUFMA; and Aldenora Bello Cancer Hospital - HCAB). Patients >18 years of age, who underwent surgery as the first treatment option and who provided informed written consent, approved by the Research Ethics Committee (CEP-HUUFMA), opinion No. 3.122.045 and CAAE No. 05918918.9.0000.5086, were included. Patients who refused to provide consent or who underwent radiotherapy or chemotherapy as primary therapy were excluded.

A survey of clinical and epidemiological data from patients with PC was performed through a review of medical records. Histological subtyping in all cases was reclassified according to the World Health Organization (2016), while the TNM system from the American Joint Committee on Cancer (AJCC), 8th Edition was used for staging. Histopathological parameters were also reviewed through re-analysis of hematoxylin and eosin (H&E) slides, which was performed independently by two pathologists (GEBS and SPCM). In cases of disagreement, the definition was established after consensus among pathologists. After reviewing the H&E slides, a formalin-fixed paraffin-embedded (FFPE) block containing at least 70% of the tumor tissue was selected for determining protein expression using immunohistochemical (IHC) methods and for polymerase chain reaction (PCR) analysis.

### HPV histological identification

Histological diagnosis of HPV was performed by identifying koilocytosis, in accordance with three main criteria described by Abadi et al. (1998), more specifically: the presence of a perinuclear halo; nuclear atypia; and presence of multinucleation.

### HPV molecular analysis

The presence or absence of HPV DNA in PC samples was performed according to a protocol described by Sambrook et al. (1989). Subsequently, nested PCR was performed in two stages. In the first, a set of generic primers, PGMY09/11, described by Gravitt et al. (2000), were used. These primers produce a 450 base pair (bp) fragment of the HPV L1 region. In the second, the primer GP5+/6+ was used, generating a 170 bp amplicon, which corresponds to the L1 region of the viral capsid. The amplicons were applied to a 1.5% agarose gel, subjected to a constant voltage of 90 V for 40 min, and visualized.

### Cyclin D1 protein expression according to IHC analysis

For IHC analysis, a rabbit monoclonal antibody anti-human-cyclin D1 (Clone EP12, Dako), purchased from Agilent (Santa Clara, CA, USA) was used. A 3-μm thick section of FFPE block was used for each case, and placed on IHC-specific slides using a pre-warmed water bath at 50°C. The slides were then heated at 70°C for 30 min for drying and removing excess paraffin tissue. Briefly, for antigen retrieval, the slides were immersed in a high pH solution (Agilent) at a ratio of 1:50 with distilled water and subjected to temperature cycling in a rinse station (PT Link PT200, Dako, Agilent Technologies), according to manufacturer’s recommendations. After antigen retrieval, endogenous peroxidase activity was blocked, and monoclonal antibody (ready to use) was applied, followed by polymer application. Diaminobenzidine (i.e., “DAB”) was then applied for 10 min to develop immunostaining, followed by counter staining with hematoxylin for 5 min. Finally, the slides were analyzed under an optical microscope.

Protein expression analysis was performed semi-quantitatively in accordance with criteria proposed by Gunia et al., (2011), which characterizes cyclin D1 expression as negative when there is an absence or weak expression of this protein only in basal cells, and positive when expressed in both basal and suprabasal cells.

### Data analysis

For statistical analysis, the chi-squared test was used to compare clinical/histopathological data for expression of cyclin D1. Survival analysis was performed using the Kaplan–Meier method to determine/estimate disease-free survival according to cyclin D1 expression, and the log-rank test was used to compare the survival curves. All analyses were performed using SPSS version 26.0 (IBM Corporation, Armonk, NY, USA). Differences with *p* ≤ 0.05 were considered to be statistically significant.

## CONCLUSIONS

Our data demonstrated that cyclin D1 is a potential biomarker for PC and is associated with poorer prognosis, although no association was found with lymph node metastasis and disease-free survival. However, due to the limited number of studies in the literature, these data reinforce the importance of performing more studies investigating the actual role of cyclin D1 in PC.

## Abbreviations

PC: Penile cancer
HPV: human papillomavirus
pRb: retinoblastoma protein
HUUFMA: University Hospital of the Federal University of Maranhão
HCAB: Aldenora Bello Cancer Hospital
HE: hematoxylin and eosin
FFPE: formalin-fixed paraffin-embedded
IHC: Immunohistochemistry
